# Deep time extinction of largest insular ant predators and the first fossil *Neoponera* (Formicidae: Ponerinae) from Miocene age Dominican amber

**DOI:** 10.1186/s12915-022-01488-9

**Published:** 2023-02-08

**Authors:** Gianpiero Fiorentino, John Lattke, Adrian Troya, Christine Sosiak, Minsoo Dong, Phillip Barden

**Affiliations:** 1 Federated Department of Biological Sciences, New Jersey Institute of Technology, Newark, USA; 2 Departamento de Zoologia, Universidade Federal Do Paraná, Curitiba, Brazil; 3 Departamento de Biología, Escuela Politécnica Nacional, Quito, Ecuador; 4 Applied Biology Program, Division of Bio-Resource Sciences, Kangwon National University, Chuncheon, South Korea; 5 Division of Invertebrate Zoology, American Museum of Natural History, New York City, USA

**Keywords:** Biogeography, Extinction risk, Dominican amber, Local extinction, Micro-CT, Random forest

## Abstract

**Background:**

Ponerine ants are almost exclusively predatory and comprise many of the largest known ant species. Within this clade, the genus *Neoponera* is among the most conspicuous Neotropical predators. We describe the first fossil member of this lineage: a worker preserved in Miocene-age Dominican amber from Hispaniola.

**Results:**

*Neoponera vejestoria* sp. nov. demonstrates a clear case of local extinction—there are no known extant *Neoponera* species in the Greater Antilles. The species is attributable to an extant and well-defined species group in the genus, which suggests the group is older than previously estimated. Through CT scan reconstruction and linear morphometrics, we reconstruct the morphospace of extant and fossil ants to evaluate the history and evolution of predatory taxa in this island system.

**Conclusions:**

The fossil attests to a shift in insular ecological community structure since the Miocene. The largest predatory taxa have undergone extinction on the island, but their extant relatives persist throughout the Neotropics. *Neoponera vejestoria* sp. nov. is larger than all other predatory ant workers known from Hispaniola, extant or extinct. Our results empirically demonstrate the loss of a functional niche associated with body size, which is a trait long hypothesized to be related to extinction risk.

**Supplementary Information:**

The online version contains supplementary material available at 10.1186/s12915-022-01488-9.

## Background

Extant communities are conspicuously shaped by speciation and migration, but a third key mechanism is only directly observable through the fossil record: extinction. Extinction plays a driving role in evolution [1, 2] and is suggested to be a key mechanism for increased evolvability in surviving lineages [3]. Extinction may be parameterized or modeled to better understand macroevolutionary processes at broad scales [4]; however, instances of local extinction and their impact on the reconstruction of lineage or community history may be otherwise unknowable without fossil evidence [5, 6]. Insular extant ecosystems such as islands offer unique opportunities to assess evolutionary processes through their isolation [7–9] but at the same time are rarely coupled with geographically contiguous fossil deposits.

The island of Hispaniola currently harbors 126 native ant species that represent an ecologically broad sample of Neotropical taxa [10]. Dominican amber, a fossil resin Konservat-Lagerstätte dated to the Miocene (~ 16 Ma; [11, 12]), is mined in the Dominican Republic and preserves over 1100 described insect species [13]. Despite inherent taphonomic and sampling biases, a total of 86 ant species have been described from Dominican amber ([14, 15]; herein). The Hispaniolan amber ant community is almost entirely modern; 84 species are placed within extant genera found in the Neotropics [14]. However, these extinct congeners suggest numerous local taxic extinctions [16]. Approximately one-third of fossil genera no longer occur on Hispaniola or the Greater Antilles but are present elsewhere in the Western Hemisphere [10, 13, 14, 16]. By comparing qualitative traits of locally extinct genera to those that are common to both fossil and extant communities, Wilson [16] suggested that size or specialized ecology may have rendered some taxa susceptible to extinction, although this hypothesis has not been tested in a quantitative framework. In the 35 years since Wilson’s study, substantially more information has been uncovered about the amber fossil and extant ant faunas, including the discovery of many new species. Here, we report a species which provides new ecological insight into taxic ant extinctions in the Caribbean.

The subfamily Ponerinae is among the most morphologically diverse and species-rich groups of ants in the world, only surpassed by Myrmicinae, Formicinae, and Dolichoderinae [17, 18]. Within this subfamily, the genus *Neoponera* stands out, presenting one of the broadest ecological and morphological radiations and widest distributional ranges among Neotropical ponerines [19, 20]. Bolton [21] synonymized *Neoponera* under *Pachycondyla*, which was later considered paraphyletic [22–25]. Mackay and Mackay [23] proposed seven species groups fitting within the current definition of *Neoponera *sensu Schmidt & Shattuck 2014. The genus currently contains 58 valid species [18].

There are more *Neoponera* species adapted to arboreal habitats than those to the ground, which is uncommon among ponerine ants (20). *Neoponera* have developed into the most ecologically diverse of all Ponerinae genera, ranging from generalist ground predators [19, 22, 26] to specialized termite raiders [27–29]. Some species even exhibit mutualistic relationships with their host plants wherein *Neoponera* workers provide herbivore protection in exchange for nesting sites and food resources, such as Muellerian bodies [19, 20, 30]. *Neoponera* is typically absent in island ecosystems, except for a few species in the Lesser Antilles [23]. Here, we describe the first fossil species of the genus and discuss its impact on reshaping the temporal and biogeographic history of the group.

## Results

### Systematic paleontology

Order Hymenoptera Linnaeus, 1758.

Family Formicidae Latreille, 1809.

Subfamily Ponerinae Lepeletier de Saint-Fargeau, 1835.

Genus *Neoponera* Emery, 1901.

*Neoponera vejestoria* sp. nov.

ZooBank LSID: urn:lsid:zoobank.org:pub:DAF246D4-5D88-4858-BDFE-6329E2507396.

### Diagnosis, worker

*Neoponera vejestoria* shows the typical diagnostic characters present in the genus, including well-developed, convex eyes placed at about head mid-length; well-developed aroliae; and slit-shaped propodeal spiracle (most species in the genus). Within *Neoponera*, this fossil mostly resembles species in the *foetida* and *aenescens* species groups. However, it is readily assignable to the *foetida* group (see more insights further below) since it shows (1) well-developed malar carinae, which are absent in the *aenescens* species group, and (2) eyes placed at about head mid-length, whereas in the *aenescens* group these are placed slightly anterad. *Neoponera vejestoria* may only be confused with three extant species in the genus, *N. dismarginata* (Mackay & Mackay), *N. carbonaria* (Smith), and *N.* ecu39704 (undescribed, see images of MEPNINV39794 on AntWeb.org). It can be separated from these taxa by the following: *N. vejestoria* shows evident striae on the body dorsum, particularly on the mesonotum and on abdominal segments III and IV (no striae on *N. dismarginata*, showing instead microfoveae; in *N. carbonaria* and *N.* ecu39794, the cuticle is almost devoid of sculpture dorsally, instead having few, usually feebly impressed striae on the meso- and metapleuron). *Neoponera vejestoria* shows blunt humeral carinae (well-developed and sharp in *N. dismarginata*; blunt to absent in *N.* ecu39794, and *N. carbonaria*). Finally, *N. vejestoria* has well-developed, apparently sharp, posterolateral nodal carinae on the petiole (absent in *N. dismarginata*; sometimes present in *N. carbonaria*, though always blunt; and always present but blunt in *N.* ecu39794). Within the *foetida* group, *N. vejestoria* is the only taxon showing a bluish-greenish iridescent cuticle. Among extant *Neoponera*, this trait has only been seen in some species of the *aenescens* group, for example, in *N. carbonaria*.

### Description, worker

*Head.* Frontal view: subquadrate, slightly longer than broad (CI 95); postocular lateral margins feebly converging posterad; posterior head margin moderately concave; eye well-developed (OI 29), convex, breaking lateral head margin, located dorsolaterally near head mid-length (OMD 0.50). In frontal/lateral view: posterior head corner convex; well-developed malar carina present, almost reaching anterior eye margin; anterior mid-clypeal margin convex; mandible with 13–15 teeth on masticatory margin, basal margin edentate; frontal lobe apparently subtriangular, slightly convex anteriorly, feebly projecting over antennal insertions, so that bulbous neck is partially visible dorsally; antennal scape relatively long (SL 119), surpassing posterior head margin by approximately three apical widths.

**Fig. 1:**
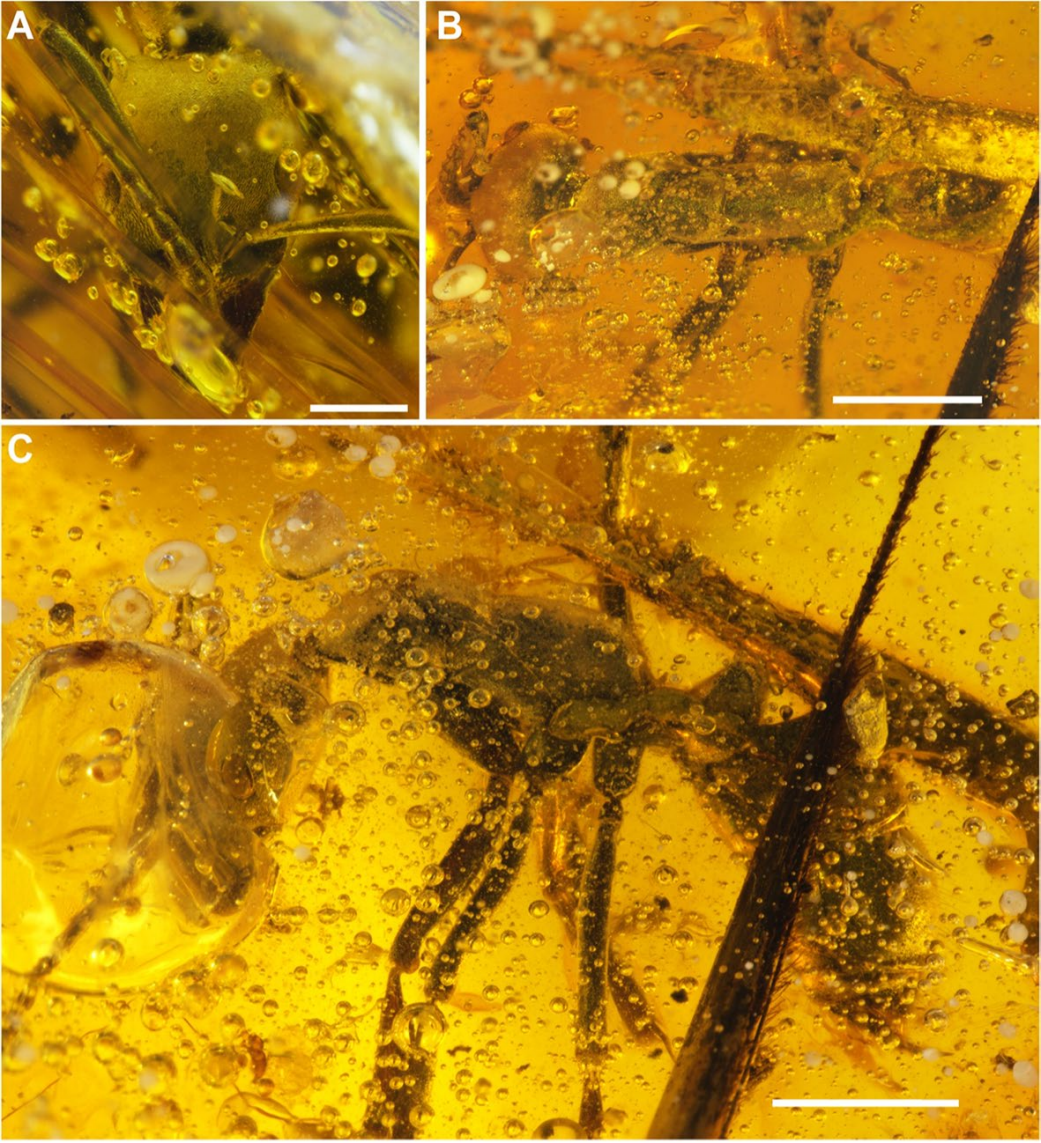
Photomicrographs of *Neoponera vejestoria* sp. nov. (Holotype, MNHNSD FOS 18.01). **A** Head in frontal view. **B** Body in dorsal view. **C** Body in lateral view. Scale bars: **A** 1 mm; **B**, **C** 2 mm

*Mesosoma*. Lateral view: dorsal pronotal margin slightly convex, humeral carina present, blunt, not salient (Additional file 1: Fig. S1A); promesonotal articulation present; mesonotum weakly convex; posterior margin sloping; notopropodeal suture present, grooved, though not deeply impressed; mesonotum broader than long (MsL = 37); dorsal propodeal margin slightly convex (Additional file 1: Fig. S1B); propodeal declivity with concave transverse section; propodeal carina with blunt elevated lateral margin; metanotal spiracle covered by lobe, propodeal spiracle slit-shaped; metapleural gland opening with seemingly reduced posterior cuticular flap (Additional file 1: Fig. S1G); probasisternum broad anteriorly and gradually narrowing posterad, grooved, with acute posterior process; mesosternal process present but reduced, apparently canine tooth-shaped: lobes are blunt distally as compared to those on metasternum; metasternal process present, fang-shaped, space between distally acute lobes greater than width of each lobe (Fig. 1C, Additional file 1: Figs. S1A and B).

Legs. Fourth metatarsus about half as long as 5th (Additional file 1: Fig. S1E); arolium well-developed, about 1/3 of claw length (Additional file 1: Fig. S1F).

Petiole. Lateral view: node sessile, higher than broad (LPI 66), subtriangular and robust, broad at base and slightly tapering atop, with anterior margin relatively straight, feebly inclined posterad, and posterior margin convex, both meeting anteriorly to nodal vertical midline; posterolateral nodal face with evident convex carina (Fig. 1C); longitudinal carina on lateral face not apparent; subpetiolar process with blunt anterior cusp. Dorsal view: subtriangular, anterior margin subacute, posterior margin straight to slightly convex.

Gaster. Prora well-developed, tooth-shaped, with subacute tip directed anteroventrad; cinctus between segments AIII–AIV well-developed (Additional file 1: Fig. S1C and Fig. S2); stridulitrum on pretergite of AIV not apparent.

*Sculpture, pilosity, and color.* Whole-body iridescent and shiny, with green/blue metallic coloration (similar to *N. carbonaria*); head with sparse erect hairs and somewhat dense short pubescence, antenna mostly devoid of pubescence, sparse erect setae present on scape and antennomeres; malar and genal regions punctate to rugulose, clypeus mostly punctate; mandibles finely punctate, covered by thick, long pilosity (mostly ventrally); dorsoposterior head surface with fine longitudinal striae; propleuron with abundant (> 10) erect hairs; mesonotum densely punctate, longitudinally striate, covered with sparse long, erect hairs (mostly dorsally); dorsal face of propodeum densely punctate, transversely striate, with sparse short erect setae and long erect setae laterally; declivity of propodeum transversely striate, with long erect setae on the lateral border. Legs with long erect and dense short appressed setae; arolium whitish; anterior face of the node with fine longitudinal striae, posterior face sparsely punctate, anterior and posterior margins with long erect setae; subpetiolar process distinctly rugulose and finely punctate; abdominal tergites III and IV longitudinally striate; gaster sparsely covered with long erect setae, lacking appressed pubescence; prora distinctly smooth and shiny; hypopygium and epipygium with long erect hairs; distinctly smooth and shiny, lacking short tooth-like setae.

Worker measurements: HW 2.39, Hl 2.54, EL 0.63, SL 2.87, OMD 0.50, ProW 1.5, WL 3.71, MsW 0.91, MsL 2.46, MfL 3.51, PW 1.08, PH 1.44, PL 0.9, AIIIL 1.41, AIIIW 1.94, AIVL 1.23, AIVW 1.77, TL 12.3; CI 95, OI 26, SI 119, MsI 37, LPI 66, DPI 120.

*Etymology*. The name is derived from the Spanish vernacular word “vejestorio,” an informal way to refer to an old person or object. The specific epithet is a feminized, non-Latinized adjective placed in apposition, thus invariant.

**Fig. 2:**
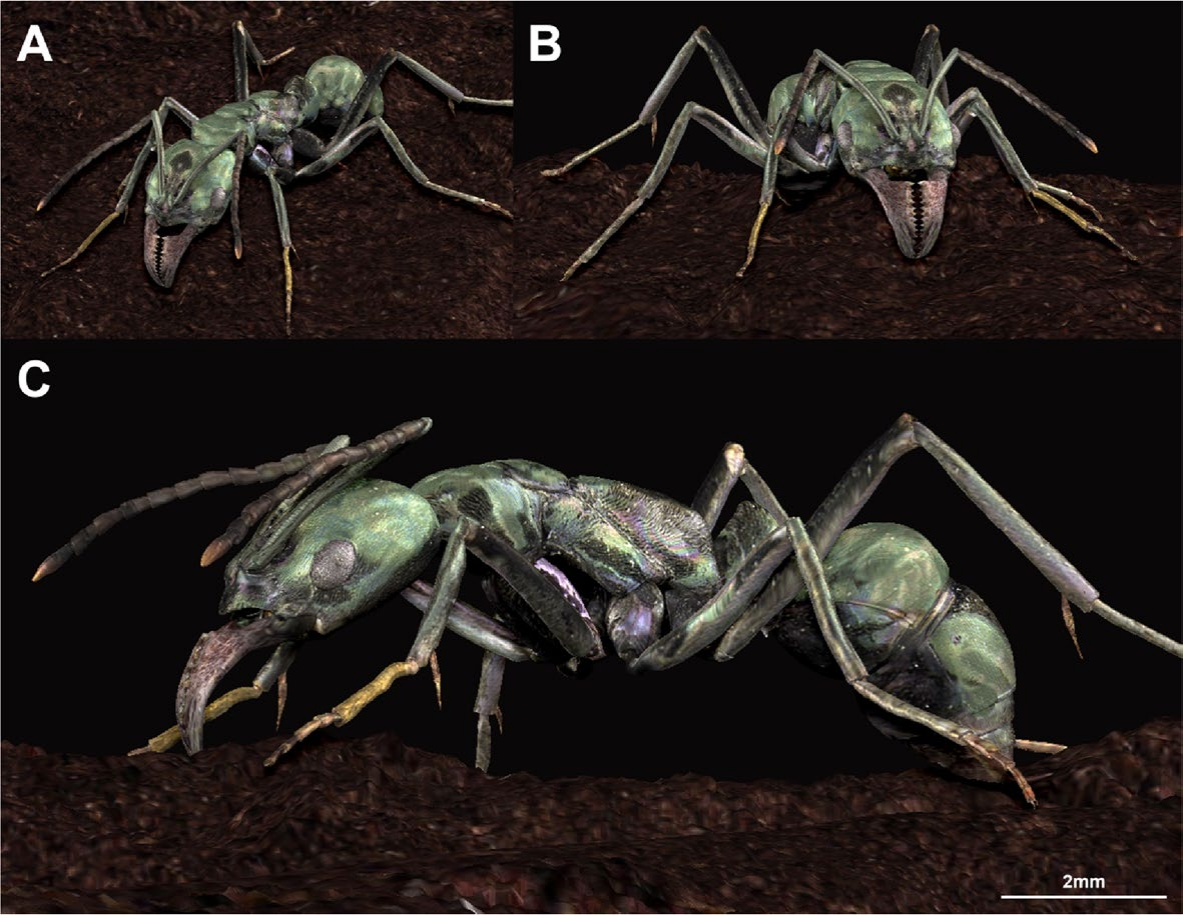
Artistic reconstruction of *Neoponera vejestoria* sp. nov. Artist: Minsoo Dong

*Type material.* Holotype. MNHNSD FOS 18.01 worker, deposited in the Museo Nacional de Historia Natural “Prof. Eugenio de Jesús Marcano,” Santo Domingo, Dominican Republic (Figs. 1 and 2, Additional file 1: Figs. S1 and S3). Preserved within a 60 mm by 40 mm section of transparent, yellow amber with abundant bubbles. Syninclusions include a long-jawed spider (Tetragnathidae), a staphylinid beetle (Paederinae), and a fungus gnat (Mycetophilidae).

*Horizon and locality*. Early Miocene, Burdigalian (ca. 16 Ma; [11]); in amber from the Northern mines of the Santiago Providence, Dominican Republic.

**Fig. 3:**
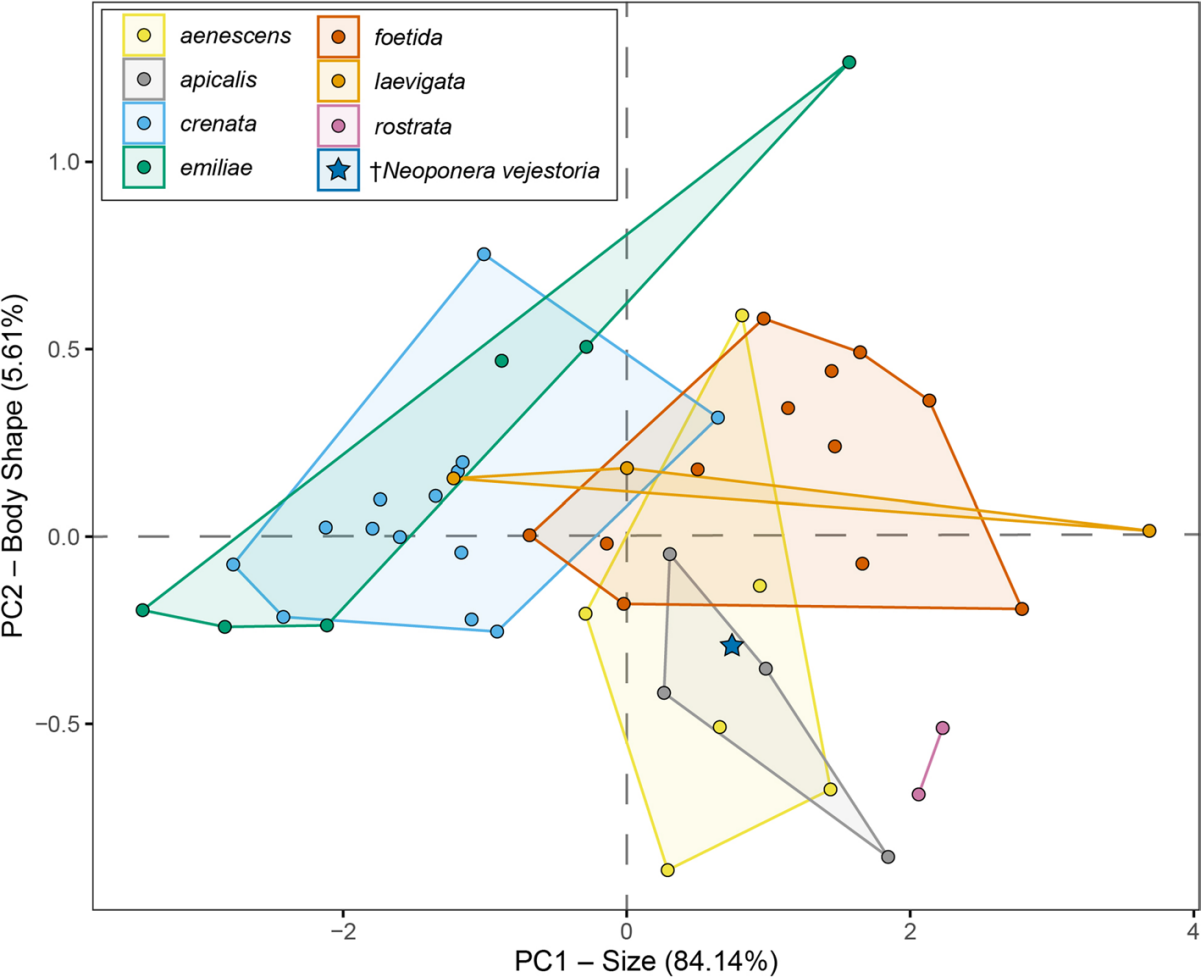
Principal component analysis morphospace of *Neoponera *ants according to species group. The sampling comprised 47 species represented by 12 linear morphological measurements. PC1 corresponds with the overall body size. *Neoponera vejestoria* is denoted with the blue star. The first five principal components of the *Neoponera* dataset make up 98% of the total variance. Principal component 1 comprises 84.14% of the variance; principal component 2 reflects the overall body shape, mainly in the pronotal width and petiolar dimensions (see Additional file 1: Fig. S4), comprising 5.61% of the variance

*Comments. Neoponera vejestoria* is the first fossil species in the genus and also the first confidently assigned to the genus level in the *Pachycondyla* genus group from the Neotropics. Morphometric analysis of 12 traits across 47 of the 58 valid *Neoponera* placed the novel species within the morphospace comprising the *aenescens* and *apicalis* species groups, but also close to the morphospace of the *foetida* group (Fig. 3). However, from a taxonomic perspective, this species is morphologically assignable to the *foetida* group, mainly due to (1) the presence of malar carinae. Though this trait presents some variation, it is a re-occurring, well-distinguished feature in all lineages of this species group. Perhaps the only exception to this rule is *N. dismarginata* where these carinae are rudimentary and hard to distinguish, but still present. As cited before, the malar carina is absent in all known lineages of the *aenescens* group (Additional file 1: Fig. S2A). (2) The eyes placed approximately on the head mid-length. This feature is among the easiest to discern in the *foetida* group and is also quite similar to the arrangement found in virtually all species in the *crenata* group, which is its putative sister clade sensu Troya et al. (unpublished). Except perhaps for *N. aenescens* (Mayr), from the nominotypical species-group, the eyes of all taxa in the *aenescens* group are placed slightly anterad on the head. Again, this feature is clearly discernible, thus easy to diagnose. (3) The humeral carinae are well-impressed in *N. vejestoria*, though somewhat blunt and not salient. This character is variable in the *foetida* group, from strongly salient and acute as in *N. foetida*, to acute but not salient as in *N. zuparkoi* (Mackay & Mackay), to blunt and feebly impressed as in *N. fisheri* (Mackay & Mackay). Nevertheless, a humeral angle is always present in all members of the *foetida* group, and it could be considered apomorphic for it, as well for all species in the *crenata* group. In contrast, the humeral carina in species of the *aenescens* group, although feebly impressed in some lineages, like in *N. aenescens* or *N. carbonaria*, is overall absent in the form of an acute border in all its members. (4) Abundant pilosity, both appressed and erect, is another remarkable feature in most members of the *foetida* group, with the exception of *N. fisheri* and *N. solisi* (Mackay & Mackay) which show much less appressed pilosity mainly on the nodal dorsum as compared to their group partners. In a similar fashion, all species in the *aenescens* group are setose, many of them show abundant appressed setae like *N. eleonorae* (Forel), but none shows abundant long setae as in members of the *foetida* group. (5) A conspicuous and grooved notopropodeal suture (Additional file 1: Fig S1A) is also present in all members of the *aenescens* and *apicalis* groups but is absent in almost all members of the *crenata* group.

Besides the three possible similar species mentioned in the diagnosis, *N. vejestoria* is perhaps also similar to *N. insignis* (Mackay and Mackay), but the petiolar node of the latter is somewhat block-shaped, approximately symmetric in lateral view. The longitudinal striae on the dorsum of the head is a putative autapomorphy in *N. vejestoria*. Only *N. foetida* (Linnaeus) and *N. theresiae* (Forel) show a similar sculpture, but this is restricted to the node and laterally on the propodeum in those species. Finally, *N. vejestoria* is easily distinguished by its notable iridescence which has been observed only in some species of the *aenescens* group, for example, in *N. carbonaria*, but in the *foetida* group it is, thus far, a novelty which warrants further research.

### Morphometric analyses

#### Species group delimitation and placement of the new species

The seven *Neoponera* species groups were relatively well-represented using sampled morphometric traits (Table 2, Fig. 3). In addition to the differences in morphology, these groups also differ in general ecology: one example being the *crenata* group, which is composed of arboreal generalists and can be readily distinguished from the *apicalis* and *aenescens* groups, which mostly are generalist epigaeic predators. This ecological variation in morphospace is unsurprising given that many of the morphological traits we sampled have been implicated in ecological occupations [31–34]. Most species ranged from medium to large in body size, with only the *crenata* and *emiliae* groups reaching significantly smaller sizes. The *laevigata*, *aenescens*, and *foetida* groups occupy similar morphospace; however, these groups are separated by additional morphological features not captured in our analysis. *Neoponera vejestoria* is a medium-sized species with morphometric affinities to the *apicalis* and *aenescens* groups but also important diagnostic features characterizing the *foetida* group.

#### Greater Antilles predator community

**Fig. 4:**
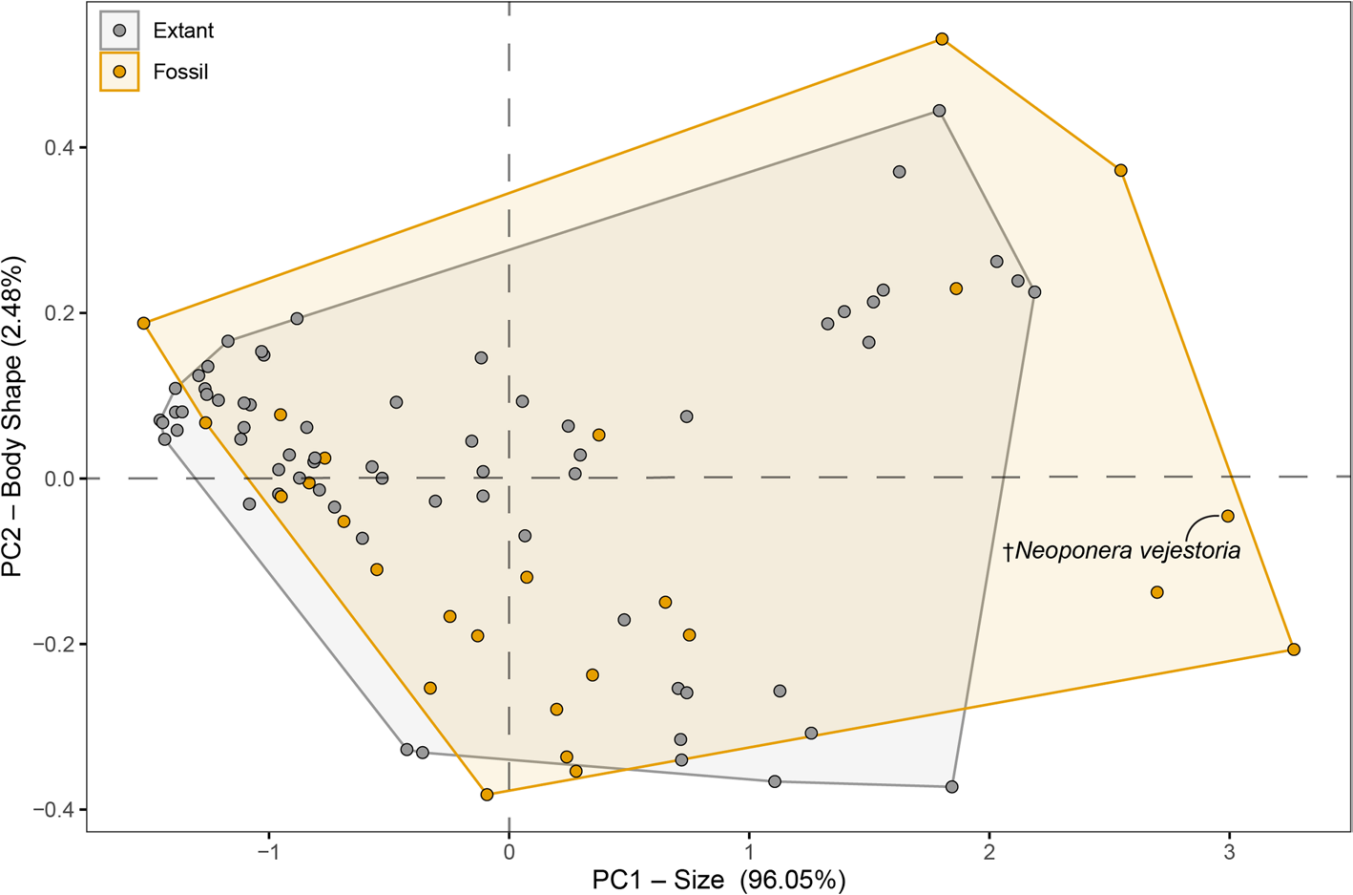
Morphospace of Hispaniolan predatory ants in fossil and extant communities. Morphometric data comprise head length, head width, and Weber’s length across 35 extant and 26 fossil species

**Fig. 5:**
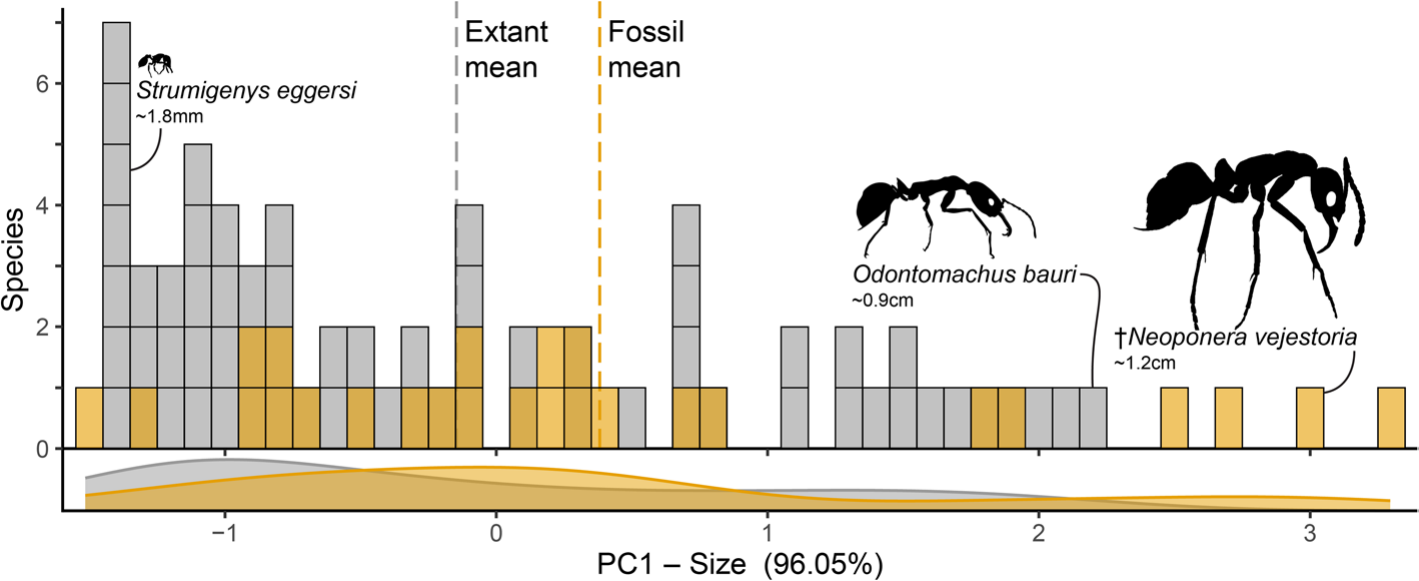
Size distribution of extant and extinct ant predators on Hispaniola. Principal component 1 derived from PCA of three morphometric traits across taxa. Fossil ants (yellow) exhibit larger body sizes than extant taxa (gray) on average and among extremes. Note: plot includes alate (queen) specimens, including the largest known fossil ant species, an undescribed *Fulakora* queen

To quantify the morphospace of predatory ants on Hispaniola, we applied principal component analyses to two separate datasets: one comprising all extant and fossil predator ants on the island and a second including only ponerine ants. In both cases, *N. vejestoria* is recovered as the largest predator ant on the island, extant or extinct (Figs. 4 and 5 and Additional file 1: Fig. S5). Overall, fossil species occupied a broader range of sizes when compared to extant species (Figs. 4 and 5). Size distribution was also more even across fossil species (Fig. 5). In contrast, extant Hispaniola ants were on average smaller, with most of the diversity clustered in the small range of sizes.

Extant ponerine species exhibit smaller body sizes on average than their fossil counterparts (Additional file 1: Fig. S5). The average distribution of both is much more even when compared to the complete Hispaniola ant dataset (Fig. 4). Body shape, as defined by our measurements, was also found to be less variable in extant ants, as opposed to the greater variability seen in fossil ants, although this is likely skewed by the large number of fossil *Odontomachus* species sampled (Additional file 1: Fig. S5).

The largest ponerines on the island are almost exclusively of the genus *Odontomachus*, except for *N. vejestoria* (Additional file 1: Fig. S5)*.* Here, we recover an extinction of larger species through time, with only *Odontomachus* species persisting as medium to large-bodied ponerine predators. However, the true variation in size of the fossil Hispaniolan ant community is likely incomplete due to the inherent sampling bias (see below).

### Predicting the ecological niche

The results of our random forest analysis predicted the nesting niche, foraging niche, and functional role of the new *Neoponera* species with out-of-bag (OOB) accuracy rates between ~ 82 and 85%. Our results indicate that *N. vejestoria* was likely a ground-nesting, epigaeic generalist predator (Table 1), in contrast to most species within the *foetida* species group, which are generally arboreal nesting predators.

**Table 1:** Predicted ecological niches for *N. vejestoria* class votes indicate the proportion of decision tree votes for the predicted ecological niche, illustrating prediction consensus

Ecological aspect	Predicted niche	Class votes	OOB error rates
Nesting niche	Ground	97.22%	15.27%
Functional role	Generalist predator	87.32%	17.10%
Foraging niche	Epigaeic	97.90%	18.14%

## Discussion

### Functional niche extinction in insular ant communities through deep time

Our results demonstrate a functional niche extinction across geographically contiguous communities through deep time. Along with a case of taxic local extinction, this niche loss is evidenced by a shift in the ecological community structure of predatory ants on the island Hispaniola. The average size of the Hispaniolan predators, both in the fossil and extant communities on the island, can be categorized as small (Figs. 4 and 5), yet the range of body sizes is broader in fossil species (Fig. 4). While aspects of our reported fossil size distribution are subject to taphonomic bias, the shift in maximum body size extremes is definitively due to local extinction of select lineages [16]. The extinction of these lineages represents more than the loss of species diversity; it also signifies the loss of functional niches common in the rest of the Neotropics. Some of these extinct niches are highly specialized. A clear example in the Caribbean is the local extinction of *Neivamyrmex* army ants [16], which are blind, nomadic predators. Specialized niche loss in this region also applies to the extinction of the trap-jaw ants *Acanthognathus* [35] and subterranean predators such as *Acanthostichus* [36].

The discovery of *N. vejestoria* provides further evidence of the molding of extant communities through the selection of certain traits. Why did this lineage undergo local extinction? Though the hypothesis that large individuals would be selected against was suggested by Wilson [16], his data were inconclusive and mostly disregarded, favoring instead selection against highly specialized species instead of body size per se. Yet, comparing the fossil *Neoponera* species to all other extant predatory species on Hispaniola, it and other locally extinct taxa are conspicuously large—*N. vejestoria* is at least one-third larger than the largest extant predatory ant (Fig. 5). Moreover, our analysis situates *N. vejestoria* as a ground-nesting, generalist predator (Table 1), in contrast to extinction-prone traits referenced by Wilson [16]. Our understanding of the true distribution of sizes in fossil insect communities is largely incomplete due to resin-capture biases inherent to amber preservation. Solórzano Kraemer et al. [37] evaluated possible sample biases using modern communities and demonstrated that insect resin preservation is contingent on distributions across forest strata; arboreal insects are overrepresented while forest floor dwelling taxa may be sampled less often. Putting this into context with the discovery of *N. vejestoria* could imply that the true range of sizes in the prehistoric ant community was perhaps even greater and could suggest additional extinct diversity among large-bodied ants in the Miocene. Another striking example of this phenomenon is the large-bodied genus *Paraponera*, which is represented in the Hispaniolan fossil ant fauna by *Paraponera dieteri* Baroni Urbani but no longer present in the Caribbean [38, 39]. Body size has been demonstrated to be a critical trait for assessing extinction risk in mammals [40, 41], fish [42], and even amphibians [43], along with most other vertebrates [44, 45]. Increased resource requirements that are associated with large size may be especially unsustainable and may render species susceptible to extinction under habitat shifts over time [43, 44]. Though this is not commonly or easily evaluated in insects, it has been demonstrated to be a potential driver of extinction in carabid beetles [46], where larger species with restricted distributions were most at risk.

Aside from *N. vejestoria*, the next largest ponerine species, either extant or extinct, belong to the genus *Odontomachus* (Additional file 5: Fig. S5). *Odontomachus* species were also the predominant large predatory species of the Greater Antilles during the Miocene and continue to be so (Additional file 1: Fig. S5). The dominance of large *Odontomachus* predators could indicate a process of niche preservation wherein all other large-bodied genera went extinct. The retention of *Odontomachus* over time could be due to unique life history traits—such as trap-jaw predation and large colony sizes—that allowed these taxa to exploit novel food resources [47–49]. *Odontomachus* and *Neoponera* are both active generalist predators occupying an epigaeic foraging niche [20]. These two genera rarely share a home range on islands. Surveys of islands such as Cocos Island [50], Barbados [51], Tobago (see [51]), and almost all the Greater Antilles record the presence [52], and sometimes the abundance of *Odontomachus*, and the total absence of *Neoponera*. Though both are reported to occur in Puerto Rico [53, 54], this report was later put in doubt [50, 51, 55]. The extinction of *Neoponera* on Hispaniola, and the current absence of the genus across the Caribbean, except Margarita, coupled with the relative abundance of *Odontomachus*, both in fossil and extant records, could suggest an opportunity for direct competition between the two lineages. This competition between *Odontomachus* and *Neoponera* may have been one of the factors, along with selection against large body size, that led to the extinction of *Neoponera* on Hispaniola.

#### A very modern ancient ant

Despite over five decades of Dominican amber fossil research, the discovery of *N. vejestoria* highlights the potential for new insight from this important Konzentrat-Lagerstätten [14, 16]. In particular, the absence of *Neoponera* from contemporary communities in the Greater Antilles [23] provides evidence for the dynamic nature of these insular ecosystems.

While distinct from its congeners, *N. vejestoria* is very similar to extant species, suggesting morphological stability through deep time in this lineage. This phenomenon is common in Dominican amber ants across the genera *Neivamyrmex* [16], *Anochetus*, *Odontomachus* [56], *Platythyrea* [57, 58], and *Cylindromyrmex* [59], among others. Schmidt and Shattuck [20] hypothesized that early *Neoponera* were likely epigaeic generalist predators. Only three species groups maintain this niche (*apicalis*, *aenescens*, and some *emiliae*) [19], while all other *Neoponera* have either become specialist predators, namely the members of the *laevigata* group, or have transitioned into an arboreal nesting niche [19, 20]. Our results here indicate that *N. vejestoria* was likely an epigaeic generalist predator, in contrast to the arboreal life observed in most of its current group partners in the *foetida* group. However, given that very little is known about the early phylogenetic and biogeographic history of *Neoponera*, it is not clear whether the predicted ecology of *N. vejestoria* represents an ancestral state or a derived state that evolved independently following its colonization of Hispaniola.

The discovery of this fossil species also provides insight into the early evolution and distribution of the Neotropical *Pachycondyla* genus group. While most ponerine ants of the *Pachycondyla* genus group (*Pachycondyla*, *Neoponera*, and *Dinoponera* among others) are some of the most abundant and dominant ants in current Neotropical ecosystems [19, 20, 23], their representation in the fossil record is lacking. Though *Pachycondyla* currently has 19 described fossil species, all of them are likely dubious and require a detailed examination [20, 60]. *Neoponera vejestoria* is a definitive fossil representative of this group, which may be incorporated into future divergence dating estimates. Schmidt [60] estimated the relative age of the most recent common ancestor (MRCA) of the genus either between 26 and 14 Ma or 24 and 12 Ma, whereas the estimated MRCA age for the *foetida* clade, as represented by a single species, *N. villosa*, was inferred by Schmidt to be around 12 Ma. Because *N. vejestoria* exhibits arguably modern morphological traits and is readily attributable to this clade, we anticipate future age estimates of the genus will change.

## Conclusions

Our report details a rare case of empirical ecological extinction in an island ecosystem evidenced by a striking amber fossil. Concomitant with the description of a new fossil species, we identify the loss of an ecological niche linked to body size, a feature long hypothesized to be linked to extinction risk. Through morphometric analysis and machine learning, we reconstruct the approximate niche of a now-extinct lineage of ants on the island of Hispaniola. Our results challenge previously proposed hypotheses regarding local extinction in this island system and demonstrate that, while this newly reported species occupies functional niches still present today, its extreme body size likely rendered it especially susceptible to extinction since the formation of the amber ~ 16 million years ago.

## Methods

### Imaging

#### Light microscopy

Details of the holotype MNHNSD FOS 18.01 were imaged with a Nikon SMZ25 stereomicroscope and DS-Ri2 camera with the NIS Elements software at the New Jersey Institute of Technology. All images are digitally stacked photomicrographic composites of several individual focal planes, which were obtained using the Nikon Elements software.

#### Micro-CT scanning and reconstruction

X-ray computed tomography scanning of the type specimen was performed at the New Jersey Institute of Technology Otto H. York Center for Environmental Engineering and Science using a Bruker SkyScan 1275 micro-CT scanner. The specimen was scanned at a voltage of 38 kV and a current of 175 μA for 70 ms exposure times averaged over 5 frames per rotation with a voxel size of 8.75 μm. *Z*-stacks were generated using NRecon (Micro Photonics, Allentown, PA) and reconstructed using 3D Slicer v4.9 [61]. A.stl file of the reconstructed 3D model was then exported for artistic reconstruction and rendering. All the reconstructed 3D models were made with Pixologic ZBrush 2021. The coloring of the model was based on the extant ant, *Neoponera carbonaria* (Smith), which has metallic coloration similar to the new fossil ant.

### Morphometric sampling

#### Similarity to extant Neoponera species

Taxonomic sampling spanned 47 of the 58 currently valid *Neoponera* species including the newly described fossil species from Dominican amber. We assigned species groups to sampled *Neoponera *sensu Troya and Lattke [62]. Standardized images were collected from AntWeb.org (www.antweb.org). Morphometric sampling included linear measurements of 12 morphological traits: five cephalic, four mesosomal, and three petiolar (Table 2, Additional file 2: Tables S1 and S2). Morphological terminology follows [23, 63, 64] for most body structures, as well as [65] for sculpture. Protocols for measurements follow those typically used in ant systematics (e.g., [66]), while incorporating additional measurements more common to Ponerinae, such as the general dimension of the petiolar node (PW, PH, PL) [64].

**Table 2:** Morphological variables used in the systematic treatment and morphometric analyses

HL	Head length. In full-face view, the maximum distance from the posterior margin of the head to the anterior margin of the clypeus
HW	Head width. In full-face view, the maximum width of the head, excluding the eyes
EL	Eye length. In lateral view, eye length is measured along its maximum length
SL	Scape length. In frontal view, the maximum length of the scape excluding the basal condyle and neck
OMD	Ocular mandibular distance. In lateral view, the distance from mandibular insertion to the anterior eye margin
PrW	Pronotum width. In dorsal view, the maximum width of the pronotum
WL	Weber’s length. In lateral view, the distance between the anterior margin of the pronotum, excluding the collar, to the posteroventral margin of the metapleuron
MsW	Mesonotum width. In dorsal view, the maximum width of the mesonotum
MsL	Mesonotum length. In dorsal view, the maximum distance from the anterior margin of the mesonotum to its posterior margin
PW	Petiolar width. In dorsal view, the maximum width of the petiole
PH	Petiolar height. In lateral view, the perpendicular distance from the posteroventral lobe of the petiolar tergite to its maximum dorsal margin
PL	Petiolar length. In lateral view, the distance between the anterior margin of the petiole, including its anterolateral projection, to its posterior margin, excluding the posteroventral folded ridge that embraces the helcium
CI	Cephalic index: HW/HL
OI	Ocular index: EL/HW
SI	Scape index: SL/HW
MsI	Mesonotum index: MsW/MsL
LPI	Lateral petiolar index: PL/PH
DPI	Dorsal petiolar index: PW/PL

#### Hispaniolan ant community structure through deep time

To assess the size distributions among fossil and extant taxa, we applied linear morphometrics to predatory ant species known to Hispaniola, which comprise 34 extant and 26 fossil species distributed across six subfamilies. Fossil predators were inferred based on the ecology of extant congeners. Given the variety of genera representing the extant predatory ant species of Hispaniola, we reduced the measurements taken to only those that have been used as a proxy for general body size in ants [67], focusing on head length, head width, and Weber’s length (Additional file 2: Tables S3 and S4). We used ImageJ [68] to obtain the measurements sourced from AntWeb.org images (2021).

### Data analysis

#### Morphospace of known Neoponera species-groups and Greater Antilles predatory ants

To assess the comparative morphospace of *Neoponera* species as well as that of the predatory species of Hispaniola, we used a dimension reduction technique, principal components analysis (PCA). We analyzed two datasets that were derived from our within-genus sampling for *Neoponera* (based on 47 of the 58 described species) and our Hispaniolan predatory ant dataset (comprising 34 extant species and 26 fossil species). We also analyzed a subset of the Hispaniolan ponerine ant data to assess how the new species compared to other Hispaniolan confamiliars. We performed all analyses in R v.4.0.3 [69] using the packages “corrplot” [70] and “FactoMineR” [71] (Additional files 3 and 4).

#### Ecological niche prediction

To estimate the likely ecological niche of the fossil *Neoponera*, we conducted a series of random forest analyses (Additional files 3 and 4). Random forest (RF) is a machine learning algorithm used for classification based on a consensus of decision trees. The algorithm partitions morphospace—derived in this case from linear measurements described above—according to a series of predefined ecological niche binnings, building a series of decision trees which each provides a “vote” on a specimen’s predicted category. The models are trained on a dataset of specimens with known ecologies: during each iteration of the model, a third of the training dataset is randomly removed and used to estimate the accuracy of that particular iteration. The converged testing error rate across all iterations of the model is considered the out-of-bag or OOB error rate for the RF model. The model’s prediction is provided as a consensus vote for each potential ecological niche occupation; for example, the nesting ecology of specimen X may be estimated as 0% carton-nesting, 94% ground-nesting, 0% lignicolous, 3% leaf litter, and 3% subterranean.

We implemented the RF models developed by Sosiak and Barden [31]. Our training data comprised the dataset from Sosiak and Barden [31] as well as measurements from species that were selected according to their ecological binning and their defined species groups. We measured a total of 28 specimens representing 13 species (1–3 specimens per species; see Additional file 2: Table S5) to include a phylogenetically relevant sample of ecomorphological diversity across *Neoponera* species groups. We then used the RF models trained on our dataset to predict the ecological occupation of *Neoponera vejestoria* sp. nov., using trait measurement data extracted from CT scan data. RF models were implemented in the R package “randomForest” [72].

## Supplementary Information

**Additional file 1: Figure S1.***Neoponera vejestoria* sp. nov. (Holotype BALDR0443): (A) Lateral view of mesosoma; (B) Dorsal view of posterior mesosoma and propodeum; (C) Lateral view of gaster; (D) Ventral vew of mesosoma; (E) Metatarsi 1-5; (F) Arolium and metatarsal claws; (G) Metapleural gland. Scale bars: (A) 2 mm, (B) 1 mm, (C) 2 mm, (D) 2 mm, (E) 0.25 mm, (F) 0.125 mm, (G) 0.25 mm. **Figure S2.** CT reconstruction of *N. vejestoria *sp. nov. to illustrate difficult to view characters. (A) Head in front view; (B) Profile view of mesosoma and gaster; Dorsal view of head, posterior mesosoma, and propodeum as a CT reconstruction (C) and as a photograph of the fossil (D). **Figure S3.** Artistic reconstruction of *N. vejestoria *sp. nov. Artist: Minsoo Dong. **Figure S4.** Representation plot for the principal component analysis morphospace of *Neoponera* ants. Sampling comprised 47 species represented by 12 linear morphological measurements. Principal component 1 (here Dim.1) is represented by all the measured traits while Principal component 2 (here Dim.2) is represented by scape length (SL), pronotal width (ProW), mesosoma width (MsW) and the petiolar dimension (PW, PH and PL).

**Additional file 2.**

**Additional file 3.** Source code for the Neoponera and Hispaniola ant community morphospace analysis.

**Additional file 4.** Source code for the Random Forest Ecological niche modeling analysis.

**Additional file 5.** Movie representation of Figure S3.

## Data Availability

All data generated or analyzed during this study are included in this published article and its supplementary information files. A movie representation of Figure S3 is presented in Additional file 5.

## Abbreviations

PCA: Principal component analysis
PC: Principal component
RF: Random forest
OOB: Out-of-bag
Micro-CT: Microcomputerized tomography
*N*: *Neoponera*
ca: Circa
Ma: Million years ago
MRCA: Most recent common ancestor
