# Supplementary material for: Deep time extinction of largest insular ant predators and the first fossil Neoponera (Formicidae: Ponerinae) from Miocene age Dominican amber
Source: BMC Biol. 2023 Feb 8;21:26. doi: 10.1186/s12915-022-01488-9 (PMC9906935; doi:10.1186/s12915-022-01488-9)
Supplement: Supplementary file 1 — Additional file 1: Figure S1.Neoponera vejestoria sp. nov. (Holotype BALDR0443): (A) Lateral view of mesosoma; (B) Dorsal view of posterior mesosoma and propodeum; (C) Lateral view of gaster; (D) Ventral vew of mesosoma; (E) Metatarsi 1-5; (F) Arolium and metatarsal claws; (G) Metapleural gland. Scale bars: (A) 2 mm, (B) 1 mm, (C) 2 mm, (D) 2 mm, (E) 0.25 mm, (F) 0.125 mm, (G) 0.25 mm. Figure S2. CT reconstruction of N. vejestoria sp. nov. to illustrate difficult to view characters. (A) Head in front view; (B) Profile view of mesosoma and gaster; Dorsal view of head, posterior mesosoma, and propodeum as a CT reconstruction (C) and as a photograph of the fossil (D). Figure S3. Artistic reconstruction of N. vejestoria sp. nov. Artist: Minsoo Dong. Figure S4. Representation plot for the principal component analysis morphospace of Neoponera ants. Sampling comprised 47 species represented by 12 linear morphological measurements. Principal component 1 (here Dim.1) is represented by all the measured traits while Principal component 2 (here Dim.2) is represented by scape length (SL), pronotal width (ProW), mesosoma width (MsW) and the petiolar dimension (PW, PH and PL). [file 12915_2022_1488_MOESM1_ESM.docx]

**Resumen**

**Antecedentes**

Las hormigas ponerinas son casi exclusivamente depredadoras y comprenden muchas de las especies de hormigas más grandes conocidas. Dentro de este clado, el género *Neoponera* se encuentra entre los depredadores Neotropicales más conspicuos. Describimos el primer miembro fósil de este linaje: una obrera del Mioceno preservada en ámbar dominicano de la Islas La española.

**Resultados**

*Neoponera vejestoria* sp. nov. demuestra un caso claro de extinción local: no se conocen especies de *Neoponera* existentes en las Antillas Mayores. La especie se atribuye a un grupo de especies existente y bien definido en el género, lo que sugiere que este es más antiguo de lo estimado previamente. A través de la reconstrucción de tomografías computarizadas y morfometría lineal, reconstruimos el morfoespacio de las hormigas existentes y fósiles para evaluar la historia y evolución de los taxones depredadores en este sistema insular.

**Conclusiones**

El fósil evidencia un cambio en la estructura de la comunidad ecológica insular desde el Mioceno. Los taxones depredadores más grandes se han extinguido en la isla, pero sus parientes existentes persisten en todo el Neotrópico*. Neoponera vejestoria* sp. nov. es más grande que todas las demás hormigas obreras depredadoras conocidas de Hispaniola, existentes o extintas. Nuestros resultados demuestran empíricamente la pérdida de un nicho funcional asociado al tamaño corporal; un rasgo que durante mucho tiempo se supuso que estaba relacionado con el riesgo de extinción.

Palabras clave: *biogeografía*, *extinción local, ámbar Dominicano, Micro CT, Random Forest*

**
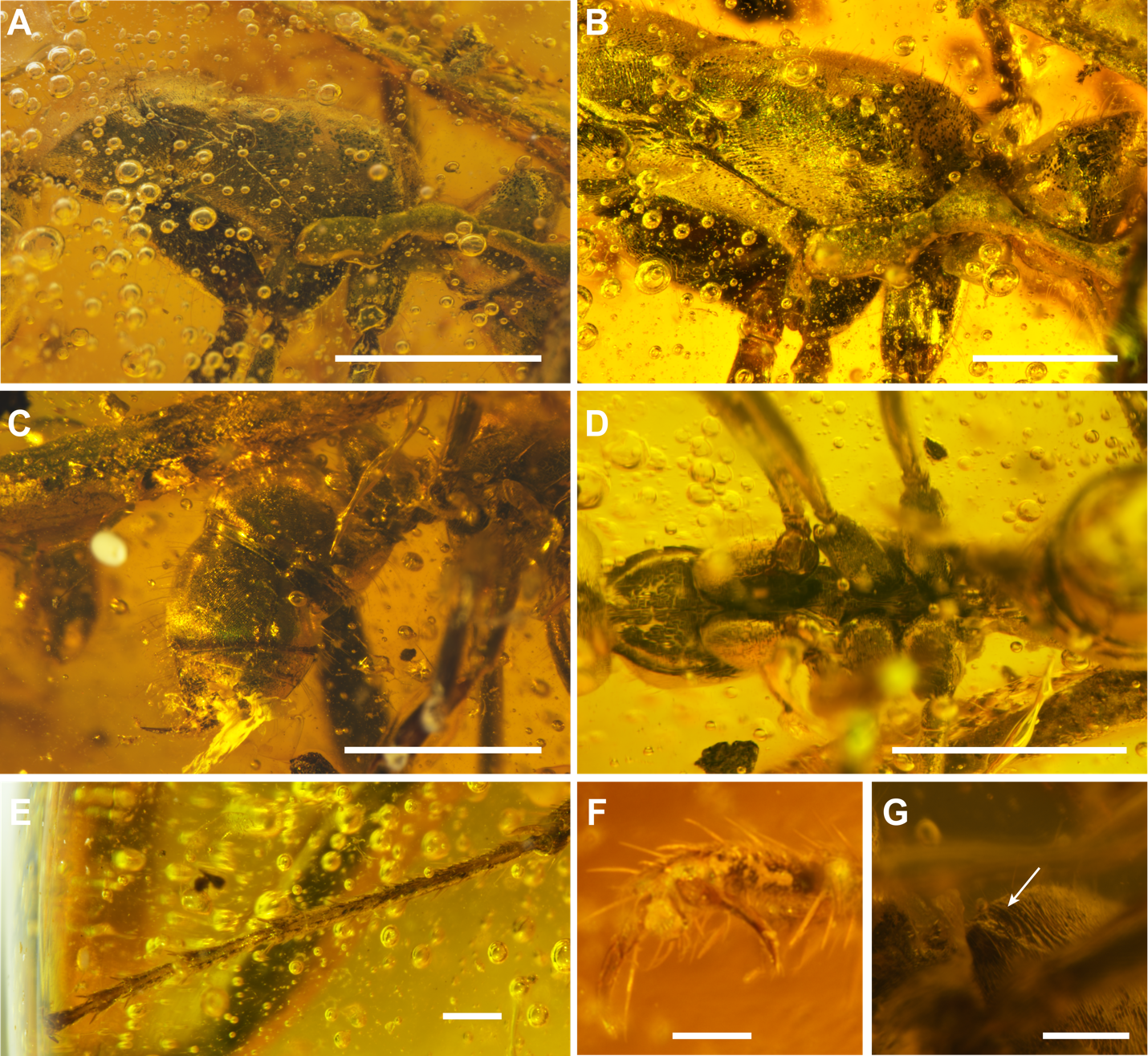
Figure S1. *Neoponera vejestoria* sp. nov.** (Holotype BALDR0443): (A) Lateral view of mesosoma; (B) Dorsal view of posterior mesosoma and propodeum; (C) Lateral view of gaster; (D) Ventral vew of mesosoma; (E) Metatarsi 1-5; (F) Arolium and metatarsal claws; (G) Metapleural gland. Scale bars: (A) 2 mm, (B) 1 mm, (C) 2 mm, (D) 2 mm, (E) 0.25 mm, (F) 0.125 mm, (G) 0.25 mm.

**
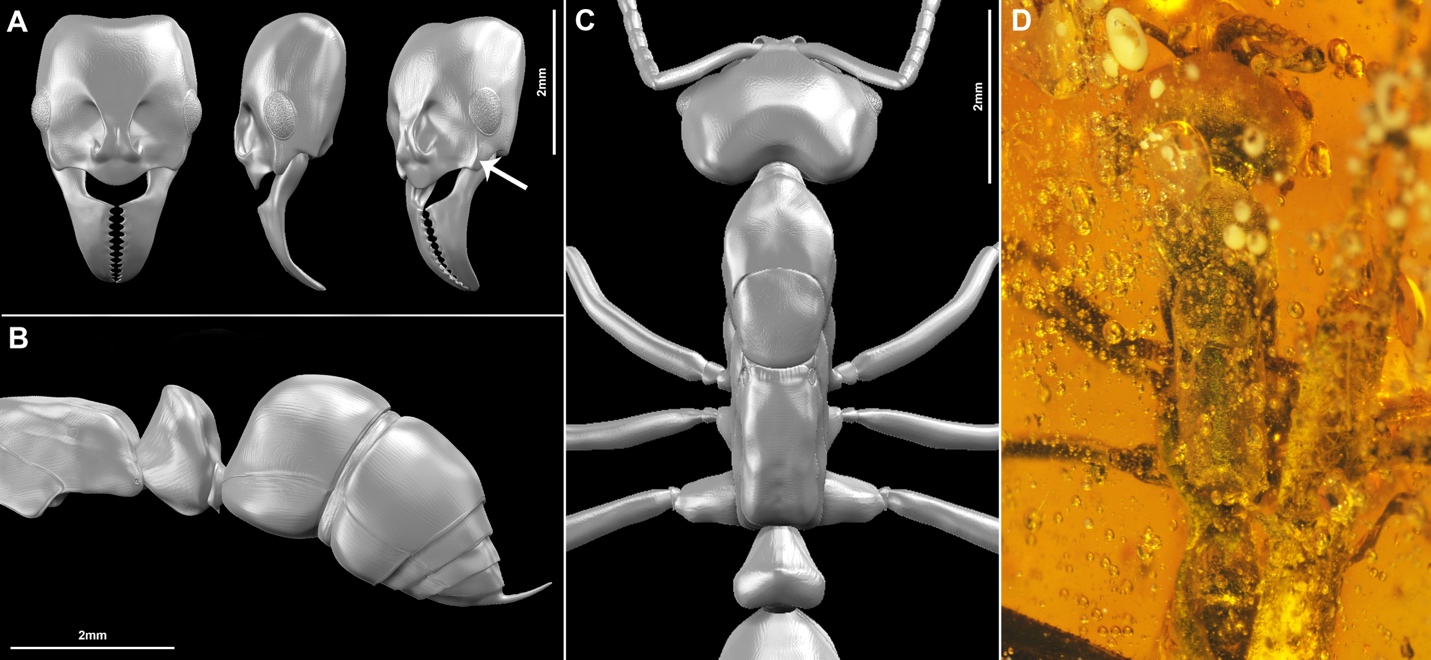
Figure S2. CT reconstruction of *N. vejestoria* sp. nov. to illustrate difficult to view characters.** (A) Head in front view; (B) Profile view of mesosoma and gaster; Dorsal view of head, posterior mesosoma, and propodeum as a CT reconstruction (C) and as a photograph of the fossil (D).


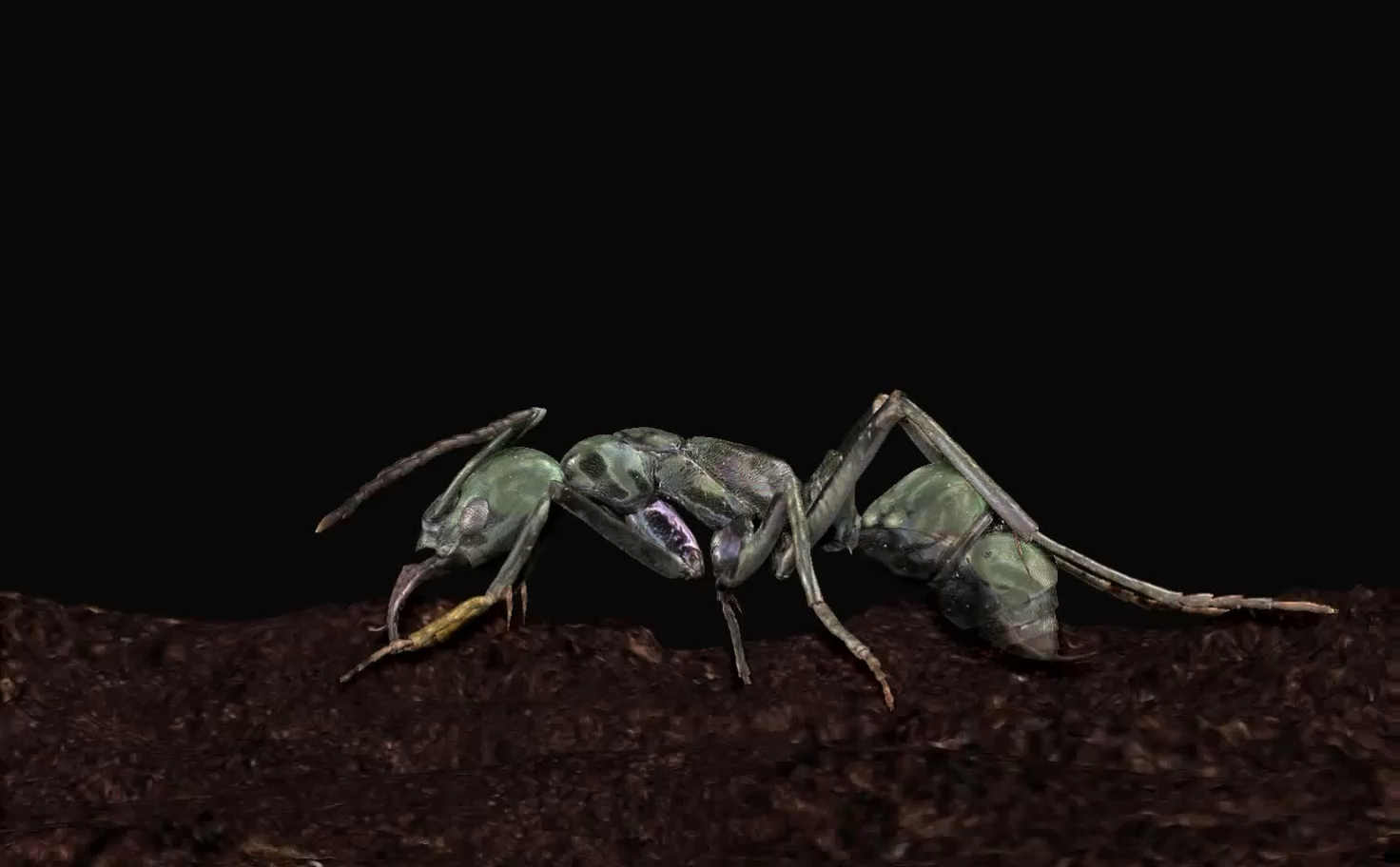


**Figure S3.** Artistic reconstruction of *N. vejestoria* sp. nov. Artist: Minsoo Dong.

**Figure S4. Representation plot for the principal component analysis morphospace of *Neoponera* ants.** Sampling comprised 47 species represented by 12 linear morphological measurements. Principal component 1 (here Dim.1) is represented by all the measured traits while Principal component 2 (here Dim.2) is represented by scape length (SL), pronotal width (ProW), mesosoma width (MsW) and the petiolar dimension (PW, PH and PL).


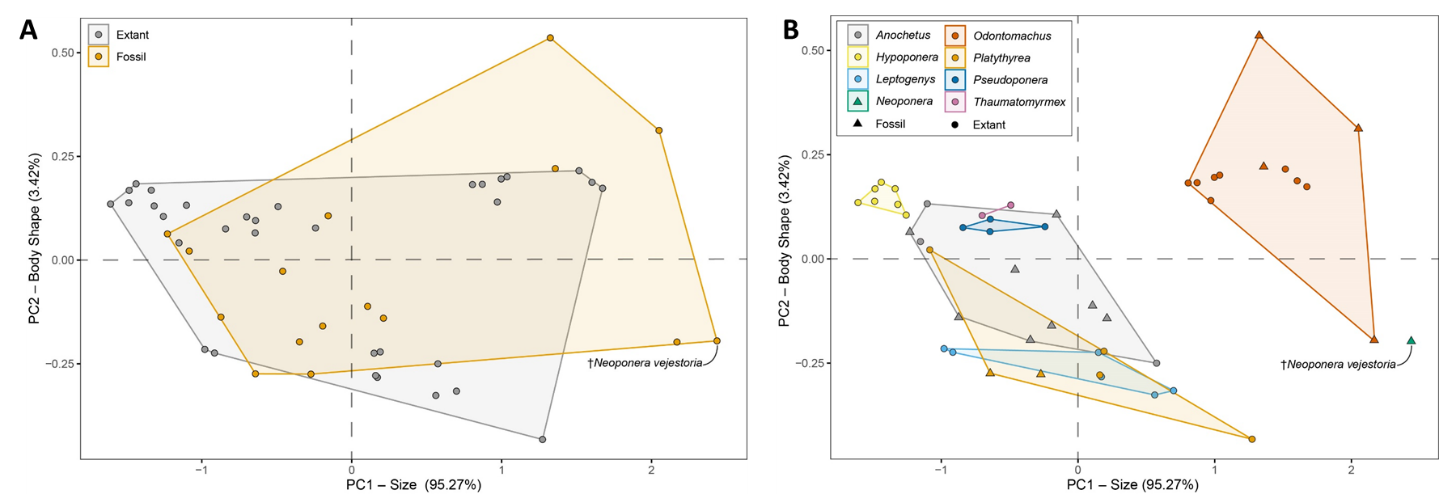


**Figure S5. Morphospace of Hispaniolan ponerine ants through time.** Sampling comprised 50 species represented by 3 linear morphological measurements. (A) Fossil ponerine ants exhibit larger body sizes. (B) The trap-jaw genus *Odontomachus* dominates the large predatory ant niche both in extant ants and in fossil ants, with only *N. vejestoria* sp. nov. exceeding the body size of this lineage on the island.
